# Supplementary material for: Sustainable Carbon Derived from Sulfur-Free Lignins for Functional Electrical and Electrochemical Devices
Source: Nanomaterials (Basel). 2022 Oct 16;12(20):3630. doi: 10.3390/nano12203630 (PMC9606865; doi:10.3390/nano12203630)
Supplement: Supplementary file 1 [file nanomaterials-12-03630-s001.zip › nanomaterials-1939134-supplementary.pdf]

Supplementary Materials

Near 2D Carbon Structures from Sulfur-free Lignins for Functional Electrical and Electrochemical Devices

Bony Thomas,[1] Mohini Sain,[1,2] Kristiina Oksman[1,2,3*]

1 Division of Materials Science, Department of Engineering Sciences and Mathematics, Luleå University of Technology, SE-97187 Luleå, Sweden
2 Mechanical & Industrial Engineering (MIE), University of Toronto, Toronto, ON M5S 3G8, Canada
3 Wallenberg Wood Science Center (WWSC), Luleå University of Technology, SE-97187 Luleå, Sweden

* Correspondence: kristiina.oksman@ltu.se; Tel.: +46-(0)920-493371

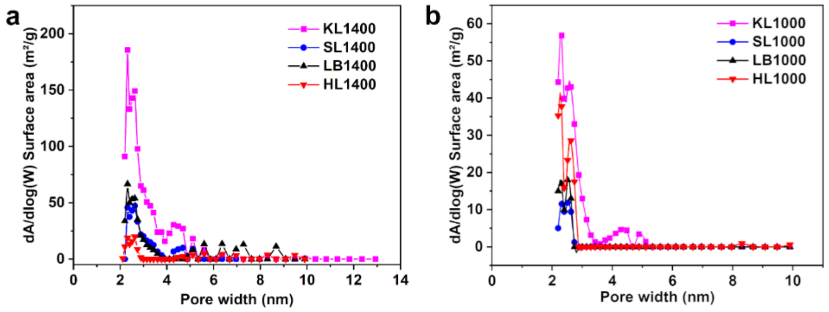

Figure S1. Pore size distribution of carbon particles obtained from DFT pore size analysis. a) carbon particles at 1400 °C and b) at 1000 °C.

Two electrode measurement

Two electrode measurement was performed using the prepared working electrodes in symmetric arrangement using Princeton Applied Research VerstaSTAT 3 Potentiostat/Galvanostat (AMETEK Scientific Instruments, Wokingham, UK). 1M H_2SO_4 was used as electrolyte. Whatman filter paper with 22 µm pore size was used as the separator. Before the measurements electrode was dipped in the electrolyte for 3 hours to completely wet the electrode with electrolyte. Capacitance was calculated using the following equation,

$$C = 4\, I\Delta t \,/\, m\Delta V$$

where C (F g−1) is the specific capacitance; I (A) is the discharge current; Δt (s) is the discharge time; ΔV (V) is the potential window; m (mg) is the total mass of electrode material.

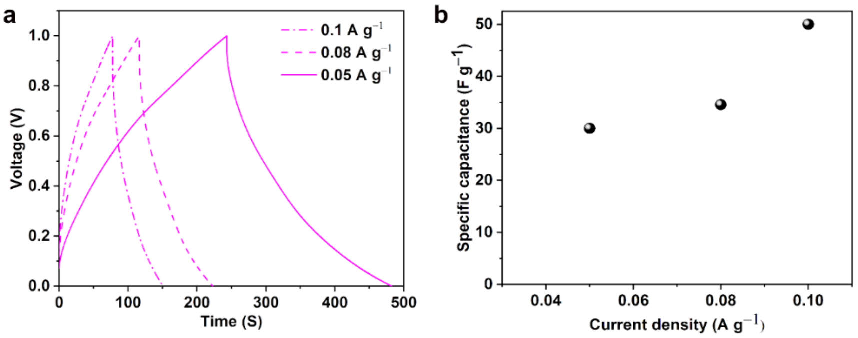

Figure S2. Results from two electrode measurement of supercapacitor. a) galvanostatic charge discharge diagrams at different current densities and b) specific capacitance obtained at different current densities.
